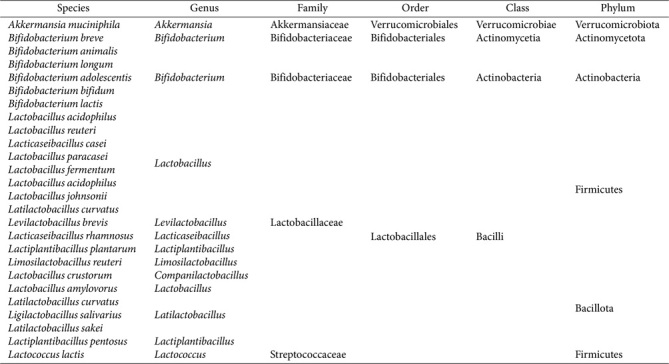# Microbiome, Potential Therapeutic Agents: New Players of Obesity Treatment

**DOI:** 10.4014/jmb.2501.01024

**Published:** 2025-04-24

**Authors:** Maqsood Ali, Navid Iqbal, Md. Abdur Rakib, Kyung-Ah Lee, Mi-Hwa Lee, Yong-Sik Kim

**Affiliations:** 1 Department of Microbiology, College of Medicine, Soonchunhyang University, Cheonan 31151, Republic of Korea; 2 Saelounbio, Seoul, Republic of Korea; 3 Nakdonggang National Institute of Biological Resources, Sangju 37242, Republic of Korea

**Keywords:** Adipogenesis, adipose tissue, browning, gut microbiome, obesity, SCFAs

## Abstract

Obesity is a global pandemic, and recent research has established a correlation between the microbiome and obesity, indicating potential treatment possibilities. This review evaluated the potential of the microbiome in treating obesity by targeting anti-adipogenesis and adipose tissue browning mechanisms. The microbiome impacts adipogenesis through lipogenesis and inflammation pathways and influences adipose tissue browning via the secretion of gut hormones and short-chain fatty acids. Understanding these mechanisms could pave the way for interventions targeting the gut microbiome to reduce obesity-related adiposity. While our understanding of the specific microbial species, metabolites, and signaling pathways involved in these processes is still limited, this review highlights the potential of microbiome-based therapies for obesity. Further research focused on identifying key microbial players and their mechanisms of action will be crucial for developing targeted and effective interventions. This will ultimately contribute to a more comprehensive understanding of obesity pathogenesis and facilitate the development of novel therapeutic strategies to combat this global health crisis.

## Introduction

Obesity is a multifaceted condition arising from an imbalance between calorie intake and energy output, associated with significant health concerns such as type 2 diabetes, non-alcoholic fatty liver disease, and specific types of cancer. The rising occurrence globally conveys a significant threat to public health, impacting both children and adults at concerning rates. In addition to the health risks, obesity imposes a significant economic burden, increasing healthcare expenses and affecting national economies [1-3].

“Anti-obesity” refers to obesity prevention, management, and treatment methods. Due to the broad knowledge of the link between obesity and health risks, even non-obese people are concerned [4]. Research suggests that exercising alone may not reduce weight. Obesity may be prevented by changing diet and exercise habits and is a significant problem due to modernization. Weight-loss drugs are popular, but they have consequences [5]. Bariatric surgery, a form of weight reduction surgery, is the most effective method for managing obesity. Due to risks and implications, this surgery is recommended only for very obese people [4]. Recently, the study of microbiome in various diseases has been highlighted and its roles in obesity gained attention and shed light on an innovative approach to managing obesity [6].

Recent research has illuminated the role of the microbiome in obesity, opening exciting new avenues for treatment. This review delves into the potential of leveraging the microbiome to manage obesity through mechanisms like anti-adipogenesis and the browning of white adipose tissue. We will explore the latest findings on how various microbiomes, both within and outside the gut, influence weight management and overall metabolic health, offering a promising new frontier in the fight against obesity.

## Adipose Tissues: Types, Components, and Their Unique Functions

Adipocytes, often known as fat cells, are specialized cells vital in storing and utilizing energy in the human body [7]. Adipocytes are the major components of adipose tissue, which functions as the principal storage and release site for energy in the body. Adipocytes are a cytoplasm rich in lipids, enabling them to effectively store and release fatty acids when required to fulfill the body’s energy requirements [8]. Understanding the role of adipose tissue in obesity requires a deep understanding of how adipocytes adapt to changes in nutrient availability. Consuming excessive amounts of high-calorie, high-fat diets can cause the enlargement of fat cells and the accumulation of fat tissue, especially in the abdomen. This can lead to insulin resistance, cardiovascular problems, and other metabolic disorders [7].

### Types of Adipose Tissue

**Fig. 1:**
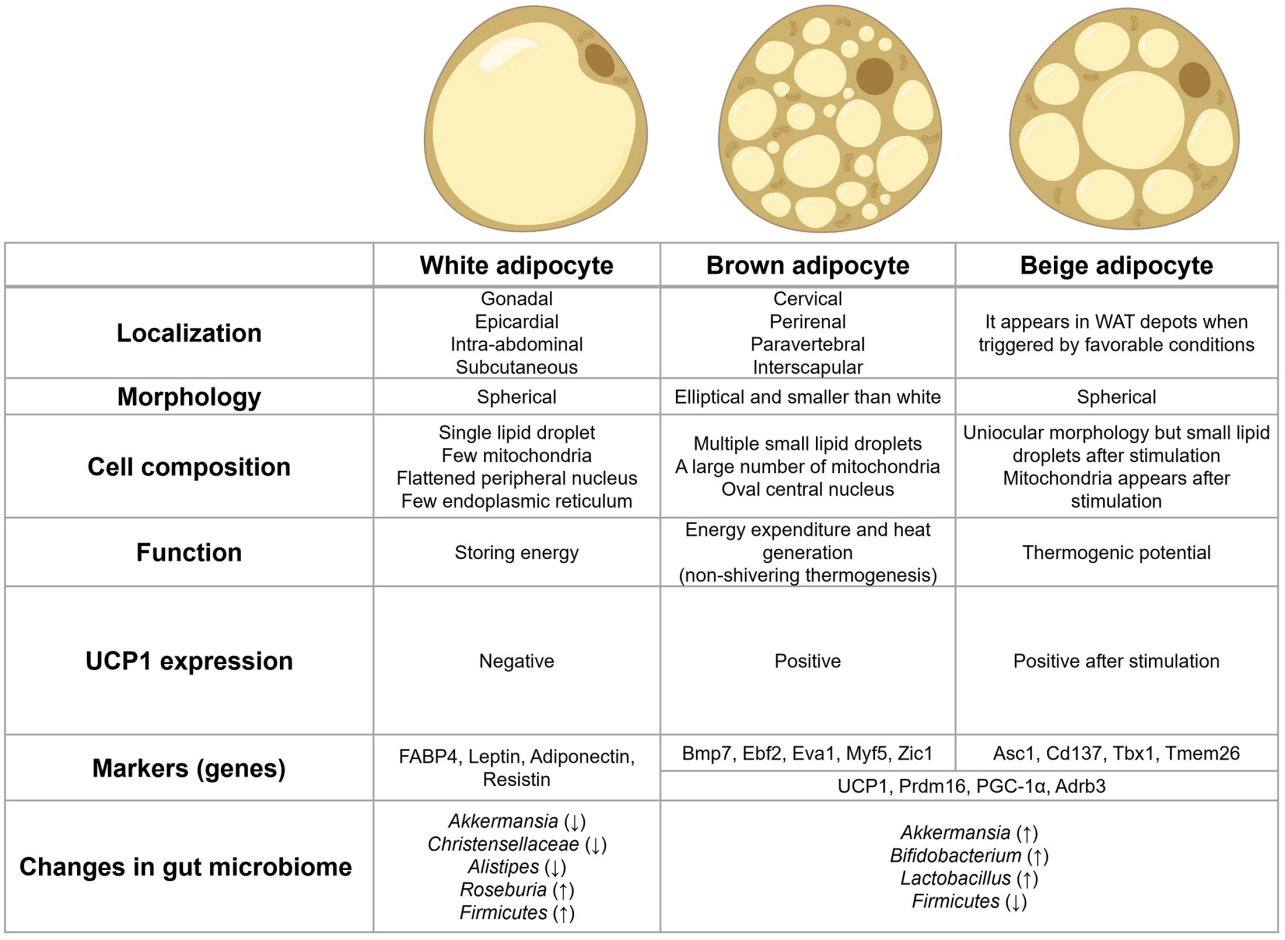
The image shows white, beige, and brown adipocytes, highlighting their unique characteristics and distinguishing features. It shows differences in the distribution of lipid droplets in the top part. Below, a table compares aspects such as primary functions (energy storage vs. heat generation), activation triggers, and molecular markers like UCP1 for brown fat, which is essential for thermogenesis. The table emphasizes the metabolic roles of WAT, particularly its function in energy storage, and the involvement of brown and beige adipocytes in heat production [10-15].

Adipose tissue, which includes beige (brite), brown (BAT, hereafter, abbreviations provided in Table 1), and white (WAT) adipose tissues, is an essential organ involved in thermoregulation and energy storage [9]. WAT stores energy as triglycerides and acts as an endocrine organ, secreting adipokines to control metabolism. Visceral WAT is associated with obesity-related conditions like dyslipidemia, insulin resistance, and systemic inflammation [8]. BAT, found in cervical, supraclavicular, and interscapular regions, comprises multilocular adipocytes with abundant mitochondria and expresses UNCOUPLING PROTEIN-1 (UCP-1), promoting non-shivering thermogenesis. When exposed to stimuli, a third type of thermogenic adipocyte, beige or “Brite,” forms in WAT [10] (Fig. 1).

**Fig. 2:**
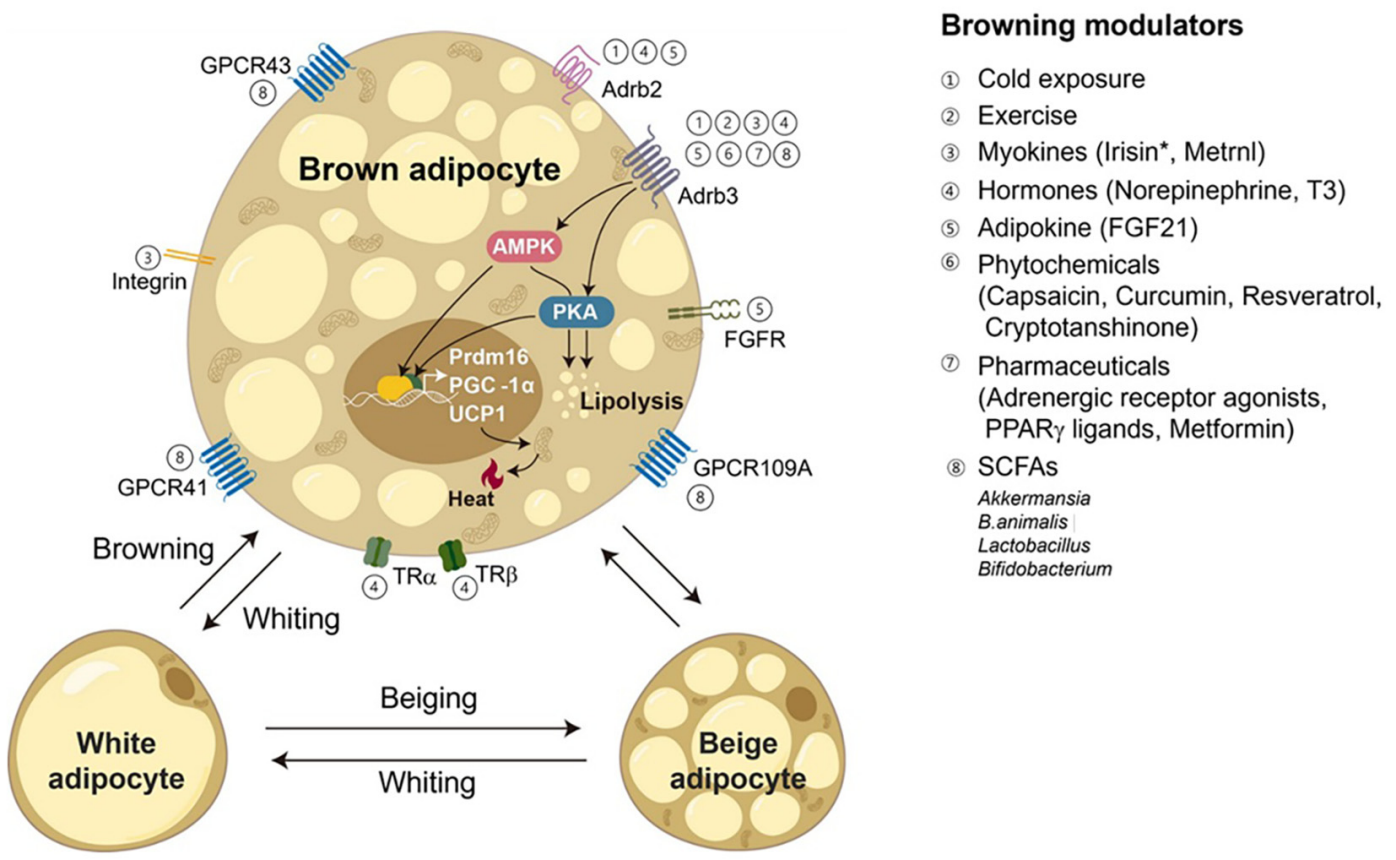
Modulation of adipocyte browning and the role of gut microbiota, cold, exercise, hormones, myokines, adipokines, phytochemicals, and medications affect browning. *Akkermansia*, *B. animalis*, *Lactobacillus*, and *Bifidobacterium* are gut microbes that can influence adipocyte browning via signaling pathways and receptors [9, 15, 16].

WAT, or white adipose tissue, is a potential therapeutic target for obesity due to its thermogenic properties similar to BAT's. Key regulators like PR/SET DOMAIN 16 (PRDM16) stimulate the formation of beige adipocytes and BAT by increasing the expression of peroxisome proliferator-activated receptor gamma (PPARγ) and ppargamma coactivator 1 alpha (PGC-1α) [11]. These regulators support BAT's vital role in thermogenesis and metabolism. PGC-1α activates mitochondrial biogenesis and UCP-1, allowing heat dissipation via an active electron transport chain (ETC) [12]. leucine-rich ppr motif-containing protein (LRP130) and nuclear pgc-1alpha (NT-PGC-1α) control mitochondrial transcription, while cell death-inducing dffa-like effector A (CIDEA) promotes lipid metabolism [13]. Surface markers like transmembrane protein 26 (TMEM26) and Tnf receptor superfamily member 9: (CD137) facilitate BAT cell identification. Understanding these pathways may lead to novel therapeutics for metabolic diseases and obesity [14]. Transcription factors like CCAAT/enhancer-binding protein beta (C/EBPβ) and PPARγ, as well as regulatory pathways like transforming growth factor beta (TGFβ) /SMAD family member 1/5/8 (SMAD1/5/8) and Wnt/β-catenin, affect lipid metabolism and cellular development [15]. Cold exposure triggers browning, triggering WAT to take on BAT-like characteristics and activating PGC-1α and PPARγ to trigger UCP1 expression. Hormonal signals like irisin and Bone morphogenetic protein 7 (BMP7) further boost thermogenesis [9]. Increasing the activity of brown and beige adipocytes can improve metabolic health and increase energy expenditure, offering promising treatment options for obesity and related metabolic disorders [16] (Fig. 2).

### Adipocyte Differentiation (Adipogenesis)

Adipogenesis, the process of creating mature adipocytes, contributes to obesity by increasing the overall number of adipocytes in the body, while obesity is caused by adipocyte hyperplasia and adipocyte hypertrophy [17].

Adipogenesis is a complex process regulated by transcription factors and signaling networks, initiated by C/EBPβ, C/EBPδ, and PPARγ, induced by insulin like growth factor 1 (IGF-1), and fibroblast growth factor 2 (FGF-2) [13]. C/EBPβ and C/EBPδ upregulate C/EBPα, a major regulator of adipogenesis, enhancing gene expression and promoting PPARγ activity and stimulating preadipocyte maturation, lipid metabolism, adipokines production, and gene activation, enhancing adipocyte differentiation through a positive feedback loop [18]. During terminal differentiation, klf transcription factor 4 (KLF4), sterol regulatory element binding transcription factor 1 (SREBP-1c), and C/EBPα become activated [19]. These substances enhance the production of lipid droplets, uptake, and gene expression specific to adipocytes [20]. As adipocytes develop, lipid droplets and indicators such as adiponectin, leptin, and Fatty acid-binding protein 4 (FABP4) increase. These indicators regulate hormones, lipid metabolism, and insulin sensitivity [15, 21]. The Wnt/β-catenin, Notch, and TGFβ signaling pathways can regulate adipocyte development and adipogenesis by regulating transcription factors. TGFβ family members modulate intracellular signaling pathways, activating SMAD1/5/8 and inhibiting SMAD2/3 pathways. Ligands of the SMAD1/5/8 pathway enhance cell proliferation, while ligands of the SMAD2/3 pathway inhibit adipocyte development [22].

### Browning of White Adipose Tissue

The process of adipose tissue browning, where WAT takes on characteristics similar to BAT, is a crucial metabolic phenomenon that has garnered considerable attention in the realms of overweight and obesity and energy balance research [23]. The browning process is primarily driven by specific biochemical pathways and gene expression changes, with UCP1, a key gene in brown and beige adipocytes, becoming more active [24]. UCP1 is responsible for decoupling oxidative phosphorylation, a mechanism that disperses energy from mitochondrial respiration as heat, rather than using it for ATP production. In brown and beige adipocytes, UCP1 disrupts this process by activating a proton channel, allowing protons to re-enter the mitochondrial matrix. This results in non-shivering thermogenesis, a phenomenon crucial for neonate animals, especially in precocial species like sheep and humans [25]. UCP1 expression and activity are regulated by various factors, including PGC-1α, which plays a crucial role in mitochondria formation and energy metabolism, and PPARγ, which stimulates thermogenesis genes. Cold exposure triggers the sympathetic nervous system to release norepinephrine, which binds to β-adrenergic receptors on adipocytes, increasing cAMP levels and activating protein kinase A (PKA) [25]. PKA phosphorylates and activates transcription factors and coactivators, leading to an upregulation of the UCP1 gene. Additional molecules like irisin and BMP7 can enhance UCP1 expression, improving thermogenic function in brown and beige adipocytes Recent research indicates that microRNAs (miRNAs) play a crucial role in regulating adipose tissue browning, a process where white fat becomes active brown fat and consumes energy. These miRNAs control genes involved in thermogenesis, adipocyte differentiation, and energy metabolism, affecting the transition of white-fat cells into brown-like ones. MiR-193b targets PRDM16, inhibiting the formation of beige adipocytes and reducing adipose tissués thermogenic capacity. Other miRNAs, miR-27, miR-30, and miR-378, influence thermogenic genes like CIDEA and UCP1 [16, 26]. Recent advancements in understanding adipose tissue browning and its molecular mechanisms present promising opportunities for developing new therapeutic strategies to combat obesity and related metabolic diseases. (Fig. 2).

## Anti-Obesity Strategies

### Inhibition of Adipogenesis

Anti-adipogenesis inhibits or prevents the formation of adipocytes and the differentiation of fat cells [27]. Mitotic clonal expansion (MCE) refers to the proliferation of preadipocytes through a limited number of mitotic divisions before cell differentiation, resulting in an augmented population of adipocytes. Activating cyclin-dependent kinase (CDK) and cyclin family proteins mediates this process. MCE is an essential stage in adipogenesis, the transformation of preadipocytes into mature adipocytes. PPARγ and C/EBPα are crucial transcription factors that facilitate adipogenesis by inducing the expression of genes specific to adipocytes and inhibition of these genes can efficiently suppress adipogenesis [28].

### Activation of Non-Shivering Thermogenesis

Thermogenesis refers to converting stored energy into heat in mammals, which occurs through shivering, characterized by muscle contractions regulated by the central nervous system, and non-shivering thermogenesis, involving heat production in BAT via UCP1 [29]. Haddish and Yun found that dopamine receptor D4 modulates lipid metabolism and both UCP1- and ATP-dependent thermogenesis in preadipocytes and muscle cells through the cAMP/PKA/p38 MAPK and cAMP/SERCA2/RYR pathways [29].

The Amp-activated protein kinase (AMPK) pathway modulates energy balance by stimulating catabolic processes and suppressing anabolic processes, thereby facilitating BAT thermogenesis, the formation of beige adipocytes, and increasing energy expenditure, while concurrently decreasing adiposity [30]. Sirtuin proteins SIRT1 and SIRT3 play a role in thermogenesis by activating PGC-1α, which is essential for mitochondrial biogenesis, and by regulating mitochondrial enzymes involved in fatty acid oxidation and oxidative capacity. Din *et al*. demonstrated that CD38 deficiency elevates the expression of SIRT1, SIRT3, and PGC-1α in muscle, thereby enhancing energy expenditure via the NAD^+^/Sirtuins pathway [31].

PPARγ modulates BAT differentiation and thermogenesis through the induction of UCP1 expression and the facilitation of lipid metabolism. Coulter *et al*. demonstrated that carotenoids augment the effects of naringenin on PPARγ and PGC-1α, thereby increasing thermogenic gene expression in human adipocytes [32].

Cold exposure enhances thermogenesis through the activation of BAT, elevation of UCP1 expression, and improvement of mitochondrial density [33]. Norepinephrine, secreted by the sympathetic nervous system, interacts with β-adrenergic receptors, resulting in heat production mediated by UCP1 through the oxidation of nutrients [33]. Portales *et al*. demonstrated that cold exposure promotes the browning of WAT, characterized by increased UCP1 expression and the presence of multilocular adipocytes [34]. McKie *et al*. demonstrated that menthol activates Transient receptor potential melastatin member 8 (TRPM8) channels, which in turn induces TRPM8- and UCP1-dependent thermogenesis in brown adipose tissue in mice [33].

### Chemicals (Phytochemicals) and Dietary Compounds-Induced Thermogenesis

Chemical and phytochemical compounds, along with dietary interventions, can enhance thermogenesis, thereby benefiting weight management and overall health. Capsaicin found in chili peppers activates transient receptor potential vanilloid 1 (TRPV1), which enhances fat oxidation and induces a brown-like phenotype in adipocytes [35]. In green tea, Epigallocatechin gallate (EGCG) inhibits the breakdown of catecholamines and modulates miRNA pathways, thereby enhancing thermogenesis [36]. Resveratrol found in grapes and red wine activates SIRT1, enhancing mitochondrial biogenesis and UCP1 expression, particularly in conjunction with exercise [37]. Berberine activates AMPK, facilitating thermogenesis via adipose tissue remodeling and the expression of UCP1 [38]. Polyphenols such as quercetin and resveratrol modulate miRNA expression, promoting the browning of WAT and enhancing mitochondrial biogenesis [39]. Fermented foods, including yogurt and kefir, enhance gut microbiome health by promoting beneficial bacteria, generating short-chain fatty acids (SCFAs), and modulating metabolic processes. These mechanisms, although promising, necessitate additional research to develop targeted dietary strategies [40, 41].

### The Role of Cytokines, Hormones, and Growth Factors in Thermogenesis

Fibroblast Growth Factor 21 (FGF21) plays a crucial role in regulating energy expenditure and thermoregulation through the promotion of WAT browning, enhancement of mitochondrial biogenesis, and facilitation of fatty acid oxidation [42]. FGF21, synthesized in the liver, WAT, BAT, skeletal muscle, and pancreas, activates cAMP/PKA signaling to promote triglyceride degradation and energy mobilization. It stimulates the PPARγ and mTORC1 pathways, leading to the upregulation of genes associated with thermogenic and mitochondrial function. In olanzapine-induced conditions, impaired FGF21 signaling leads to decreased UCP1 levels, reduced BAT temperature, and diminished thermogenic gene expression due to dysregulated H3K9me3 [42].

*Irisin*, a myokine secreted by skeletal muscles, plays a crucial role in thermogenesis and energy metabolism. It activates BAT, induces WAT browning, and stimulates UCP1 expression, leading to increased energy expenditure [43]. Irisin activates PGC-1α, upregulates Fibronectin type iii domain-containing protein 5 (FNDC5) expression, and enhances mitochondrial biogenesis and fatty acid oxidation through AMPK activation. *Enteromorpha prolifera* polysaccharides (EPP) can improve insulin signaling and energy metabolism in obese mice [44].

Bone *Morphogenetic Proteins* (BMPs) are involved in thermogenesis, regulating cellular differentiation and suppressing it by inhibiting WAT browning and reducing brown adipocyte activity [45] and downregulate gene expression and promote white adipocyte maintenance [45]. However, BMP inhibition promotes thermogenesis by activating genes and converting white adipocytes into beige/brite ones. Cold exposure or pharmacological interventions can downregulate BMP signaling, stimulating adipocyte browning, potentially presenting a therapeutic target for obesity and metabolic disorders [46].

Interleukins (ILs) regulate adipose tissue function and energy homeostasis. IL-6 and IL-1β recruit and activate brown and beige adipocytes, promoting thermogenesis. IL-6 stimulates gene expression, transforming WAT into metabolically active beige-like tissue. IL-1β induces WAT browning, enhancing energy expenditure and glucose homeostasis [47].

## Gut Microbiome: New Players in Obesity

### Microbiome

**Fig. 3:**
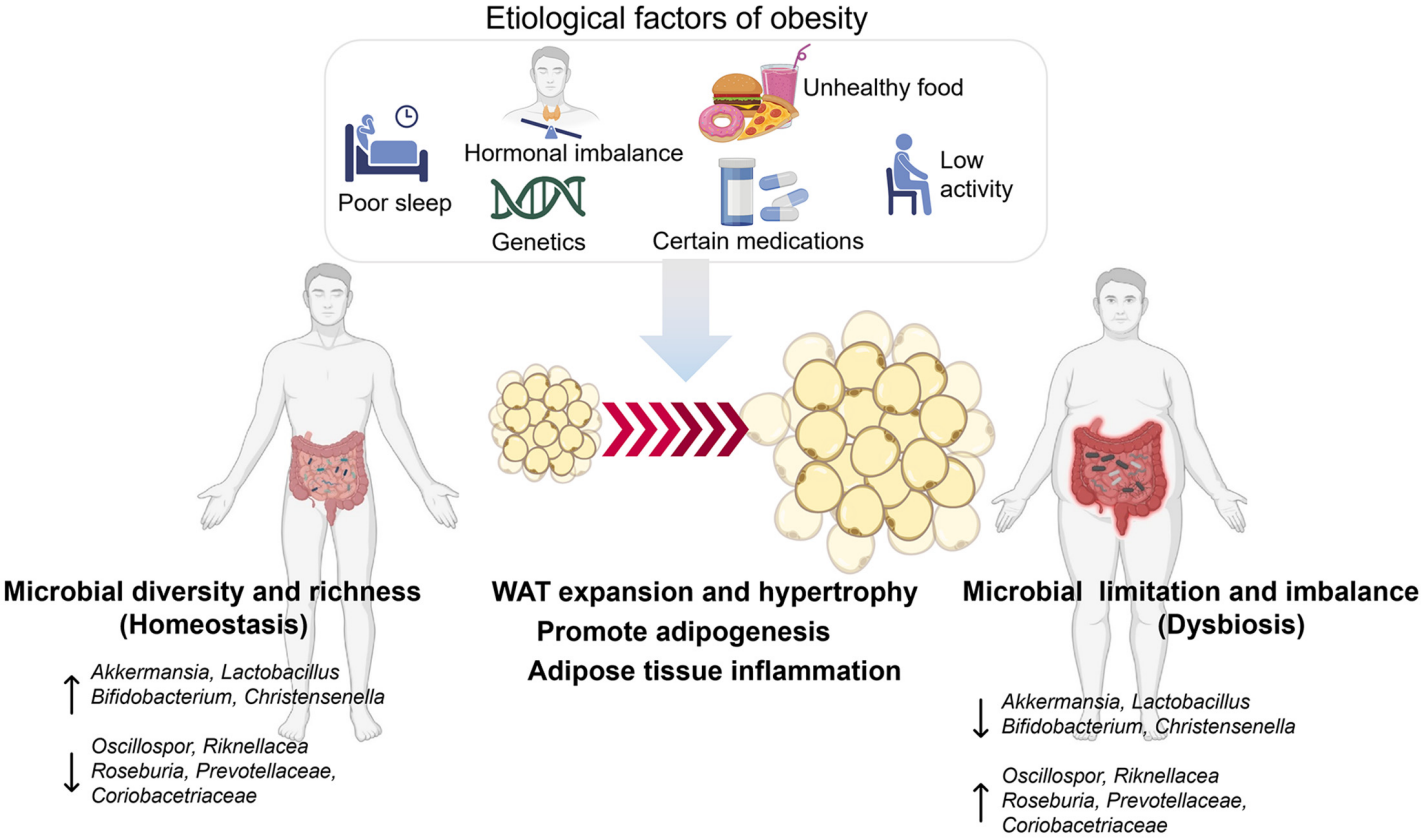
The link between gut bacteria, lifestyle factors, and adipose tissue development, which leads to obesity and inflammation. The core route depicts the evolution of WAT from normal size to expansion and hypertrophy, which promotes adipogenesis and inflammation. The first part discusses contributory variables such as inadequate sleep, hormone imbalances, high-calorie foods, sedentary behavior, heredity, and drugs. On the left, a healthy person with a balanced gut microbiome is illustrated, with bacterial diversity and richness contributing to homeostasis. On the right, a person with dysbiosis (gut microbial imbalance) is depicted, indicating that limited microbial diversity is associated with obesity and inflammation. Key microbial species such as *Akkermansia* and *Lactobacillus* are cited in both healthy and dysbiotic conditions, implying a function in metabolic health and illness [48-52].

The gut microbiome, a complex ecosystem of microorganisms in the human gastrointestinal tract, has garnered significant scientific attention in recent years [48]. Research indicates an intricate link between gut microbiome, human metabolism, and the development of health conditions like obesity. The human digestive tract contains 10 trillion microbiotas that regulate metabolism, nutrient absorption, and immune response. Dysbiosis has been linked to neurological, inflammatory, and metabolic disorders, and understanding the interaction networks between microbiomes and hosts can offer new disease treatments [49]. The gut microbiome significantly influences obesity treatment by regulating energy homeostasis, metabolic processes, and the immune system, regulating energy expenditure, dietary energy extraction efficiency, and SCFA generation, which control appetite and energy consumption [50]. Obesity and gut microbial ecology are closely linked, with normal-weight individuals having higher *Bacteroidetes* and lower *Firmicutes* than obese individuals. The gut microbiota comprises *Firmicutes* and *Bacteroidetes*, comprising 70-90% of the population [51, 52]. Recent research has shown a positive association between obesity and the *Firmicutes*: *Bacteroidetes* (F/B) ratio in humans. However, this relationship may not be universally applicable across different populations. Obese individuals often have a higher F/B ratio compared to lean individuals, with studies showing an increase in body mass index (BMI) and a positive correlation with metabolic markers. Dysbiosis, an imbalance in gut microbiota, is linked to obesity[53]. A predominance of *Firmicutes* over *Bacteroidetes* is associated with metabolic disorders, suggesting this ratio may influence obesity development. A study in Croatia found no significant association between the F/B ratio and excess body weight, suggesting age and gender may play a more critical role in obesity than the F/B ratio itself [54]. The relationship between the F/B ratio and obesity may be influenced by various factors, including diet, lifestyle, and genetic predispositions [54]. Further research is needed to clarify these dynamics and their implications for obesity management. *Akkermansia muciniphila*, an anaerobic gut bacterium, has been identified as a key player in the gut microbiome, with potential therapeutic applications for obesity and metabolic disorders, including improved gut barrier function [55] (Fig. 3).

### Microbiome in Anti-Adipogenesis

Recent studies show that gut microbiota, specifically *A. muciniphila*, can prevent adipogenesis and obesity in mice and reduce body weight, adipose tissue mass, and adipocyte count in mice fed a high-fat diet. This effect is believed to be due to the suppression of genes involved in adipogenesis and activation of AMPK signaling [56]. The molecular mechanism of treating obesity with *A. muciniphila* using integrated multiomics techniques. Results showed that the treatment reduced lipid accumulation, downregulated mRNA expression of adipogenesis proteins, and downregulated proteins involved in fat cell development, fatty acid digestion, and adipocyte energy metabolism, as well as adipocyte metabolites related to glycolysis, the TCA cycle, and ATP [57]. The treatment of 3T3-L1 cells with *A. muciniphila* cell lysate led to a decrease in lipid accumulation and a decrease in mRNA expression of adipogenesis and lipogenesis proteins [20]. Researchers studied the impact of live and pasteurized *A. muciniphila* strains on inflammatory responses, and lipid, and glucose metabolism in obese mice. They found that both strains reversed high-fat diet-induced metabolic dysregulation and obesity, stopped weight increase after one week, reduced primary adipose tissue weight, adipogenesis/lipogenesis, serum total cholesterol, improved glucose control, and reduced inflammation. They also restored gut architecture and liver structure in HFD-damaged rats [58]. Probiotics have shown potential in anti-obesity therapy, as demonstrated in an in vitro adipogenesis experiment. *Bifidobacterium longum* subsp. *infantis* YB0411 promotes adipogenic differentiation in 3T3-L1 preadipocytes, reduces triglyceride accumulation, and lowers p62 and LC3B levels. It also inhibits AMPK, reducing body weight and fat accumulation in mice with high-fat diet-induced obesity [59]. YBS1, a *Lactobacillus acidophilus* DS0079 supplement, has been found to inhibit adipocyte development by regulating the p38 MAPK/PPARγ signaling pathway. This suggests it could be a promising supplement for tackling obesity. YBS1 decreases 3T3-L1 cell growth, builds triglycerides, and activates key fat cell development genes like PPARγ, adipocyte fatty acid binding protein 4, and adiponectin. This suggests YBS1's potential in combating obesity [60].

*Lactobacillus reuteri* *J1* has been found to treat obesity by regulating lipid and glucose metabolism, reducing fat mass, dyslipidemia, glucose homeostasis, and insulin sensitivity. The bacteria alter gut microbiota and bile acid composition, inhibiting the ileum FXR-FGF15 pathway and promoting FXR-SHP, ultimately affecting the browning of WAT in obese mice [61]. *Lactobacillus plantarum* LMT1-48 demonstrated significant anti-obesity effects in mice fed a high-fat diet and induced with *Enterobacter cloacae*. These effects included reduced body weight, fat volume, leptin levels, and total cholesterol. The study also observed increased gut microbiota diversity, specifically in *Verrucomicrobia* and *Proteobacteria*, with LMT1-48 administration [62]. The study found that a combination of *Limosilactobacillus reuteri* and caffeoylquinic acid (CQA) can reverse high-fat diet-induced obesity in mice. Oral CQA supplements increased adipose tissue browning and improved metabolic performance. However, CQA therapy improved anti-obesity benefits by altering gut flora. Both treatments increased thermogenesis, but supplementing with propionate from an external source replicated the weight-reducing effects [44].

*In vitro* studies have demonstrated that certain strains of gut microorganisms possess the capability to inhibit adipogenesis and modify lipid metabolism. In a study, it was observed that *Lactobacillus rhamnosus* GG (LGG) and *Bifidobacterium animalis* subsp. *lactis* Bb12 (Bb12) had a decreasing effect on adipogenesis in 3T3-L1 preadipocytes [63]. According to a study, certain strains of *Lactobacillus plantarum* were able to inhibit adipogenesis in 3T3-L1 cells. This was achieved by reducing the expression of adipogenic genes, including PPARγ and C/EBPα [64] (Box 1)(Table 2)

### Microbiome in White Adipose Tissue Browning

Cold temperatures significantly impact the gut microbiome, reducing the prevalence of certain bacterial phyla like *Bacteroidetes*, *Proteobacteria*, *Tenericutes*, *Actinobacteria*, *Verrucomicrobia*, and *Cyanobacteria*, while promoting the growth of *Firmicutes* and *Deferribactere*, according to various studies [65]. The first group of bacteria’s decreases is attributed to their inability to thrive in low-temperature environments, while the latter group’s increase is believed to be due to their higher adaptability to cold stress [66]. Transplantation of “cold microbiota” into germ-free F mice may enhance insulin sensitivity, improve cold tolerance, and promote fat loss. UCP1, PRDM16, PGC-1α, and PPARγ levels are elevated in BAT and subcutaneous WAT. The research indicated that mice exhibiting enhanced BAT thermogenesis and altered plasma bile acid profiles demonstrated improved glucose tolerance and reduced body fat [67]. Recent studies show conflicting results regarding the effects of antibiotics or germ-free mice on WAT browning. Cold stress depletes gut microbiota, inhibiting UCP1-dependent thermogenesis. Butyrate partially restores this effect, suggesting it regulates appropriate thermogenic responses. However, alternative macrophages, inflammatory cells, and type 2 cytokine signaling do not affect thermogenesis or energy expenditure in GF mice. The scientists hypothesize that these contradictions could be due to factors like living conditions, dietary nutrients, rodent age, sex, and antibiotic administration methods [68].

The study on *Lactobacillus* and *Bifidobacterium* bacterial culture supernatant (BS) found that BS promotes white-fat browning and lipolysis in vitro and in vivo, suggesting that *B. longum* DS0956 and *Lactobacillus rhamnosus* DS0508 could be beneficial supplements for obesity and metabolic disease [56]. Park *et al*. found that the probiotic bacteria *Lactobacillus amylovorus* KU4 (LKU4) increased body temperature, mitochondrial levels, and thermogenic gene program in mice on a high-fat diet. LKU4 released RIP140, which stimulated UCP1 expression and browned WAT by enhancing PPARγ and PGC-1α interaction. LKU4 also elevated plasma lactate, affecting the interaction between PPARγ and PGC-1α, and preventing diet-induced obesity [69]. Mahmoud *et al*. investigated synbiotics' potential to reduce diet-induced obesity by increasing thermogenic genes and causing brown-like phenotype in WAT. They found synbiotics improved lipid profile, liver enzymes, insulin sensitivity, and BAT gene expression in high-fat rats [70] (Box 1) (Table 2).

### Changes in Gut Microbiome for Treatment of Obesity

**Calorie restriction (CR)**. Caloric restriction (CR) is a method that helps prevent obesity by limiting daily calorie intake while maintaining healthy eating habits. It reduces weight, fat, hyperglycemia, hyperinsulinemia, and inflammation. CR also alters gut microbiota diversity, improving metabolism and longevity in various creatures. It increases beneficial bacterial taxa like *Bacteroidetes* and *Akkermansia* while decreasing harmful ones like *Firmicutes*. This alteration is believed to contribute to CR's metabolic advantages [71]. Studies show that reducing CR can reduce inflammation and metabolic dysfunction while modifying gut microbiota composition and functional capability. However, low-calorie diets are unsustainable, as 80% of individuals gain weight and have metabolic disorders after returning to normal calorie intake. Post-obesity microbiota is linked to reduced energy expenditure and flavonoid production [72].

**Intermittent fasting (IF)**. IF is a dietary pattern that involves fasting and eating. It has been extensively studied for health conditions like chronic pain and has shown effects on metabolic, cardiovascular, immune, and neurobiological systems [73]. The gut microbiome, crucial for overall health, can be influenced by IF. The fasting method being studied has the potential to modify gut bacteria by influencing circadian rhythms, metabolism, and lifestyle factors like sleep. These changes could potentially have an impact on health outcomes, as the gut microbiome is crucial for maintaining overall health. Therefore, IF could potentially alter the composition and function of the gut microbiota, leading to various health benefits [74]. Studies have investigated various patterns of IF, such as time-restricted feeding, alternate-day fasting, and periodic fasting [75]. Time-restricted feeding is a dietary method that limits food intake to a specific time frame, typically 8 to 12 hours, with the remaining hours dedicated to fasting [76]. Alternate-day fasting is a dietary strategy that alternates between regular caloric intake and significant caloric restriction or complete fasting days [77]. Periodic fasting involves extended, usually several-day periods of fasting, after which individuals resume their regular eating habits [78]. Different IF patterns can affect the gut microbiome, with time-restricted feeding promoting beneficial bacteria abundance and reducing pathogenic ones [79, 80]. Alternate-day fasting has been linked to an increase in bacteria that promote metabolic health, including *A. muciniphila* [6, 81]. Prolonged fasting may have unclear effects on the gut microbiome, with some studies suggesting reduced microbial diversity and increased bacteria utilizing alternative energy sources. These changes improve metabolism, immunity, and well-being while increasing beneficial gut flora like *Bacteroidetes* and *Lactobacillus* species and reducing *Firmicutes* and *Proteobacteria*, which cause inflammation and obesity [82].

**Bariatric surgery.** Bariatric surgery, particularly Roux-en-Y gastric bypass and sleeve gastrectomy, can significantly alter the gut microbiome, potentially leading to weight loss and improved metabolic health. The surgery reduces *Firmicutes*, such as *Lactobacillus*, *Clostridium*, *Faecalibacterium*, and *Roseburia*, and increases *A. muciniphila* and *Proteobacteria*, improving insulin sensitivity and glucose balance. Additionally, it decreases the diversity of SCFA-producing species, crucial for maintaining gastrointestinal health [83]. For instance, a study found increased levels of *Lactobacillus* species post-surgery, which correlated with improved metabolic health markers [84]. Another investigation highlighted substantial changes in bacterial groups such as *Bacteroides* and *Fusobacterium*, linking these alterations to reductions in BMI and enhanced glucose metabolism [85]. Furthermore, the interplay between gut microbiota and the immune system post-surgery has been shown to influence liver health, with significant reductions in inflammatory markers observed [86]. However, the complexity of these interactions suggests that not all patients experience uniform changes in their microbiome, indicating a need for personalized approaches in post-bariatric care [87].

**Ketogenic diet (KD).** The KD is a dietary strategy involving high-fat and low-carbohydrate intake, promoting ketosis, a metabolic state linked to weight reduction and improved metabolic well-being [88]. Reducing carbohydrate intake through KD decreases populations of beneficial bacteria such as *Bifidobacterium*, which are linked to anti-inflammatory properties and the maintenance of gut health. It also decreases butyrate-producing *Firmicutes* species and reduces fecal concentrations of SCFAs, which are essential for energy metabolism, immune regulation, and the health of colonic epithelial cells [89]. The modifications may result in immediate weight loss by adjusting energy utilization and minimizing fat accumulation. Nonetheless, the long-term effects of KD present concerns, such as diminished microbial diversity and overall bacterial counts, which may jeopardize gut barrier function and heighten the risk of chronic diseases [90]. The association between KD and the *Firmicutes*-to-*Bacteroidetes* ratio, a biomarker associated with obesity, continues to show variability across research findings. Probiotic supplementation aimed at *Bifidobacterium* or *Akkermansia* may help address these microbial changes; however, there is a lack of extensive long-term safety data. Researchers demonstrated KD boosts good bacteria like *A. Muciniphila* and *Lactobacillus* species in the gut and reduces *Escherichia* and *Streptococcus* bacteria [91, 92].

### The Role of SCFAs

**Fig. 4:**
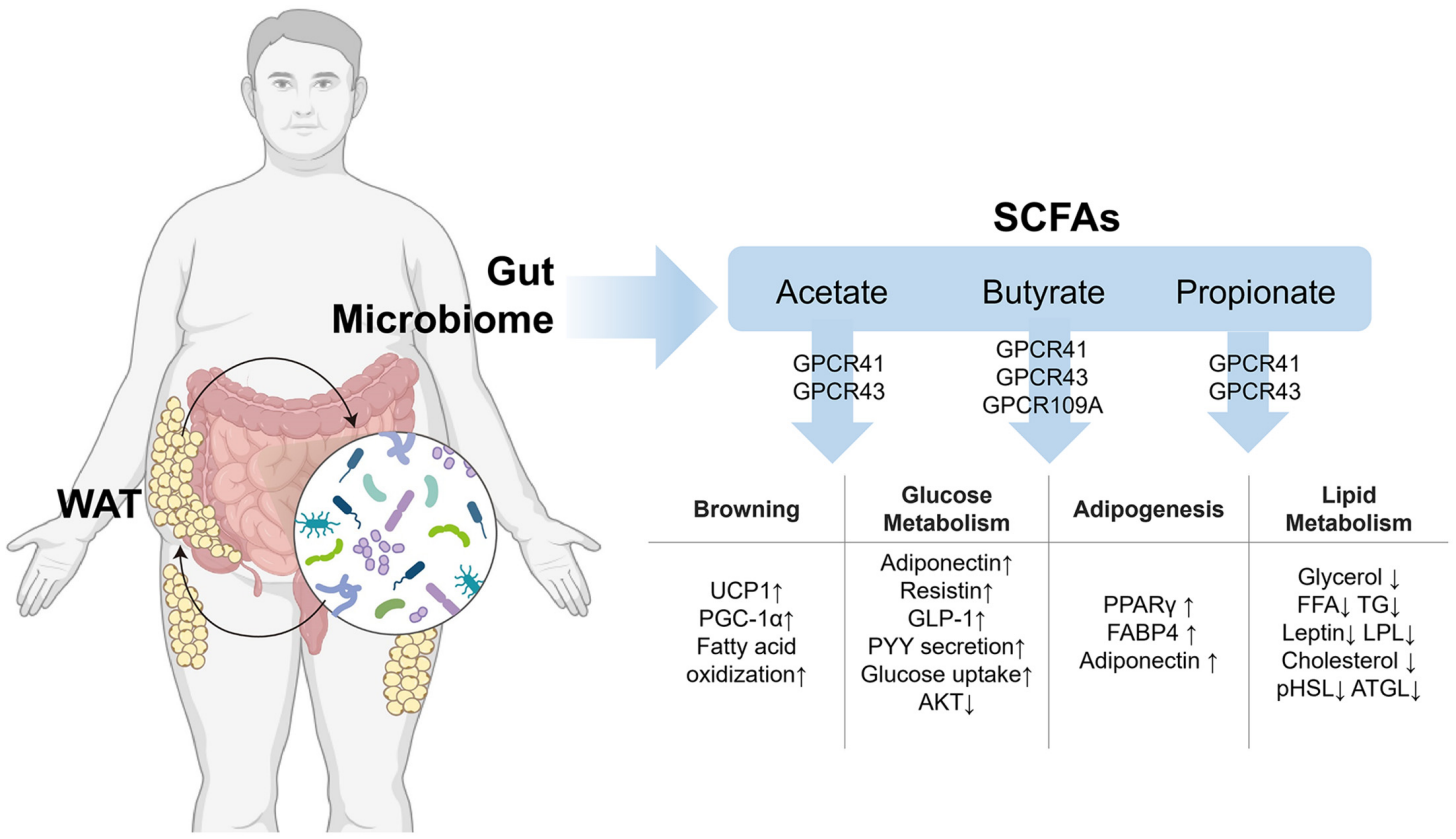
Studies show that SCFA affects nutritional status and energy balance in WAT by signaling via browning, glucose metabolism, adipogenesis, and lipid metabolism. SCFAs play a crucial role in adipose tissue metabolism and inflammation, regulated by GPR41, GPR43, and GPCR109A in adipose tissue and immune cells. These receptors control metabolic and inflammatory processes, affecting adipogenesis, browning, and energy expenditure. Propionate and acetate suppress adipogenesis, while butyrate increases lipid synthesis and insulin sensitivity. In addition, bile acid receptors FXR, TGR5, and PRDM16 contribute to adipose tissue thermogenesis [96-98, 101].

SCFAs, produced by gut microbes through dietary fiber fermentation, can regulate immune responses, energy metabolism, and adipogenesis by downregulating genes, and their production is influenced by diet, gut microbiota composition, and host genetics [93] Key SCFAs include acetate, propionate, and butyrate, synthesized by bacterial strains such as *Bifidobacterium*, *Bacteroides*, *Clostridium*, *Faecalibacterium prausnitzii*, *A. muciniphila*, and *Lactobacillus fermentum* which are crucial for maintaining gut balance and providing therapeutic benefits [94, 95] (Fig. 4).

Butyrate, a prominent SCFA, enhances gut health by serving as an energy source for colonocytes and reducing inflammation. It inhibits adipogenesis by suppressing adipogenic genes such as PPARγ and activates AMPK, improving insulin sensitivity and increasing energy expenditure [96]. It also modulates lipid metabolism through the activation of SREBP-1c and GLUT4 [97]. Butyrate activates GPR109A, eliciting anti-inflammatory effects, promoting fat oxidation, and improving insulin sensitivity, highlighting its potential as a target for obesity treatment [98].

Propionate supports thermogenesis by stimulating the browning of WAT and activating GPR41 and GPR43, increasing UCP1 expression and energy expenditure [99]. Similarly, acetate regulates adipogenesis by inhibiting transcription factors like PPARγ and C/EBPα and reducing lipid metabolism enzymes such as FAS and Lipoprotein lipase (LPL), thereby preventing preadipocyte maturation [100]. Their influence on PRDM16 further enhances the thermogenic activity of BAT and WAT browning. Recent studies show that dietary interventions can modulate SCFA production and gut microbiota composition. For example, seabuckthorn polysaccharide supplementation increased SCFA-producing bacteria like *Bifidobacterium* and *Bacteroides*, reducing body weight and improving lipid profiles in high-fat-diet-induced obese mice [101]. Similarly, combining tea polyphenols with *L. rhamnosus* R5 elevated SCFA levels and enriched beneficial bacteria like *A. muciniphila*, improving lipid metabolism and reducing obesity [102]. SCFAs represent a promising avenue for obesity management by influencing adipogenesis, thermogenesis, and energy metabolism, highlighting the importance of diet and gut microbiota in therapeutic strategies.

## Clinical Trials

Recent studies have explored the link between gut microbiota and obesity, with various procedures including the administration of probiotics, prebiotics, and synbiotics, and fecal microbiome transplantation (FMT) emerging as viable approaches to address the imbalances in the microbiome associated with obesity. This has led to increased interest in treating obesity and metabolic diseases [103] (Table 3).

### Fecal Microbiome Transplantation (FMT)

FMT, a technique that involves transplanting gut microbiota from a healthy individual to someone with an impaired microbiome, has emerged as a potential therapeutic approach for treating obesity and metabolic disorders. This involves transferring fecal matter from a healthy donor to a recipient, and modifying the gut microbiome to improve metabolic health. Numerous studies have investigated the efficacy of FMT in treating obesity and metabolic syndrome. Animal models have shown favorable results in improving metabolic parameters, inhibiting weight gain, and mitigating metabolic complications in obese mice when cohabitating with lean mice [104].

FMT has shown positive effects on metabolic health in human studies, with two randomized controlled trials showing improved peripheral insulin sensitivity and lower HbA1c levels in patients receiving donor FMT compared to placebo [105]. A study found that transplanting gut microbiota from obese to lean individuals led to a significant increase in body fat production, primarily due to the proliferation of certain bacterial taxa, suggesting that gut microbiota can influence energy extraction and storage [106]. Clinical studies have explored the use of FMT as a potential treatment for obesity, with one study showing enhanced insulin sensitivity and decreased body weight in obese individuals with metabolic syndrome transplanted from lean donors [107].

### Probiotics, Prebiotics, and Synbiotics in Obesity Treatment

A pilot study on overweight or obese insulin-resistant volunteers found that daily oral intake of live or pasteurized 10^10^ *A. muciniphila* bacteria for three months was safe and well-tolerated. Pasteurized *A. muciniphila* significantly improved insulin sensitivity, reduced insulinemia and plasma total cholesterol, and reduced body weight, fat mass, and hip circumference compared to the placebo [108].

A randomized control design assessed the effects of probiotic interventions containing specific strains of *Bifidobacterium*, *Lactobacillus*, *LeviLactobacillus*, *Lacticaseibacillus*, and *LigiLactobacillus* on gut microbiota and metabolic outcomes. The study involved 56 postmenopausal women who were obese and randomly divided into three groups: one group received a daily probiotic dose of 2.5 × 10^9^ CFU, another group received a daily probiotic dose of 1 × 10^10^ CFU, and the third group received a placebo. Probiotics can alter the bio-physiological function of the initial and end microbiota, but not its taxonomic framework [109]. A study on weight loss patients found that synbiotics supplement, consisting of probiotics and prebiotics, altered gut microbiota composition, promoting beneficial bacteria species. The study found that synbiotics supplements may modify bio-physiological function but not the taxonomic structure of the initial and final microbiota, affecting body composition measures and obesity biomarkers. The study was placebo-controlled [110].

A clinical trial by Sung *et al*. found that *Bifidobacterium breve* B-3 (BB-3) effectively reduced adiposity, reducing waist and hip circumferences compared to a placebo group. The waist-to-hip ratio decreased but was not statistically significant after 12 weeks. No adverse effects were observed. The study suggests that BB-3 is a safe and effective approach for body fat reduction, making it a viable option for weight loss [111]. A study evaluating the impact of *L.. plantarum Dad*-13 probiotic powder on gut microbiota and intestinal health in overweight adults found that both probiotic and placebo groups had similar pH levels and lipid profiles before and after intake. The treatment group decreased weight and BMI, increased *Firmicutes*, and increased *Bacteroidetes*, particularly *Prevotella* [51]. The study investigated the impact of *L. plantarum* *K50* (LPK) on body fat and lipid profiles in obese individuals. After 12 weeks, both groups showed similar body weight, fat mass, and abdominal fat region. The LPK group showed a decrease in total cholesterol, triglycerides, leptin levels, and increased *L. plantarum*, while the placebo group experienced an increase in triglycerides. The intervention also led to a decrease in *Actinobacteria* [112]. A randomized clinical study by Hadi *et al*. found that consuming synbiotics significantly reduced body weight, total cholesterol, triglycerides, low-density lipoprotein cholesterol, stress, anxiety, and depression compared to a placebo group. However, the study did not affect HDL-C, SBP, DBP, FPG, fasting insulin, BMI, or WC. The study suggests that synbiotics supplementation can improve TG, TC, LDL-C, body weight, stress, anxiety, and depression in overweight or obese individuals [113].

A randomized, double-blind, placebo-controlled research examined 72 overweight people. The probiotic groups took 1 × 10^10^ colony-forming units of *Lactobacillus curvatus* HY7601 and *Lactobacillus plantarum* KY1032, whereas the placebo group had the same product without probiotics for 12 weeks. The probiotic group saw greater improvements in body weight, visceral fat mass, waist circumference, and adiponectin than the placebo group. HY7601 and KY1032 supplementation increased *Bifidobacteriaceae* and *Akkermansia*ceae and decreased *Prevotellaceae* and *Selenomonadaceae* in gut microbiota [114] (Table 3).

## Conclusion and Future Prepositives

The microbiome is a key factor in obesity development and management, offering potential therapeutic interventions. Its complex interaction with adipose tissue, adipogenesis, browning, and the microbiota provides a multidimensional approach to obesity control. Current approaches, such as suppressing adipogenesis and stimulating browning through cold exposure, drugs, and dietary components, offer promising opportunities. However, the microbiomés increasing significance presents novel opportunities through its impact on adipogenesis and thermogenesis processes. As research advances, these methodologies will be crucial in developing more efficient anti-obesity therapeutics.

● A critical strategy involves creating personalized interventions that fit an individual's gut microbiome composition, understanding how it varies among healthy individuals, changes with age, and is influenced by factors like nutrition, medicine, ethnicity, location, and lifestyle, while also considering factors like age.

● Advanced technologies like shotgun metagenomics are needed for further investigation of bacterial strains, including their functions and metabolomics.

● To fully understand the benefits of probiotics in managing body weight, thorough investigations beyond genus classification are necessary. Baseline evaluation of gut microbiota and longitudinal monitoring of individual responses are crucial in clinical investigations. Combining different approaches can enhance intervention effectiveness and optimize outcomes for obesity patients.

● The relationship between gut microbiome and obesity is still evolving, and comprehensive research, including large-scale clinical trials, is needed to validate the efficacy and safety of microbiome-based interventions in obesity treatment.

● Biomaterial-based therapies have shown potential in targeting and regulating the gut microbiome to address obesity-related issues. These therapies offer personalized, long-lasting solutions for managing gut microbiome dysbiosis and improving weight management. As our understanding of the gut-microbiome-obesity relationship grows, biomaterial-based therapies are expected to play a crucial role in restoring metabolic balance and mitigating obesity-related complications.

## Figures and Tables

**Table 1:** Abbreviations

Name	Memo	Name	Memo
AMPK	Amp-activated protein kinase	GLP-1R	Glucagon-like peptide 1 receptor
BMI	Body mass index	GLP-1	Glucagon-like peptide 1
BMPs	Bone morphogenetic proteins	IL	Interleukins
BAT	Brown adipose tissue	IF	Intermittent fasting
CR	Calorie restriction	KD	Ketogenic diet
C/EBPβ	CCAAT/enhancer-binding protein beta	PPARγ	Peroxisome proliferator-activated receptor gamma
C/EBPα	CCAAT/enhancer-binding protein alpha	PGC-1α	PPAR gamma coactivator 1 alpha
C/EBPδ	CCAAT/enhancer-binding protein delta	PRDM-16	PR/SET Domain 16
CFU	Colony-forming unit	PKA	Protein kinase a
cAMP	cyclic adenosine monophosphate	SCFAs	Short-chain fatty acids
CQA	Cytosine-caffeoylquinic acid	SIRT	Sirtuin
FXR	Farnesoid x-activated receptor	SMAD1/5/8	Smad family member 1/5/8
FMT	Fecal microbiota transplantation	TGFβ	Transforming growth factor-beta
FGF21	Fibroblast growth factor 21	UCP1	Uncoupling protein-1
GPCR	G protein-coupled receptors	WAT	White adipose tissue

**Table 2:** In vitro and In vivo indicate the role of microbiome in the anti-obesity.

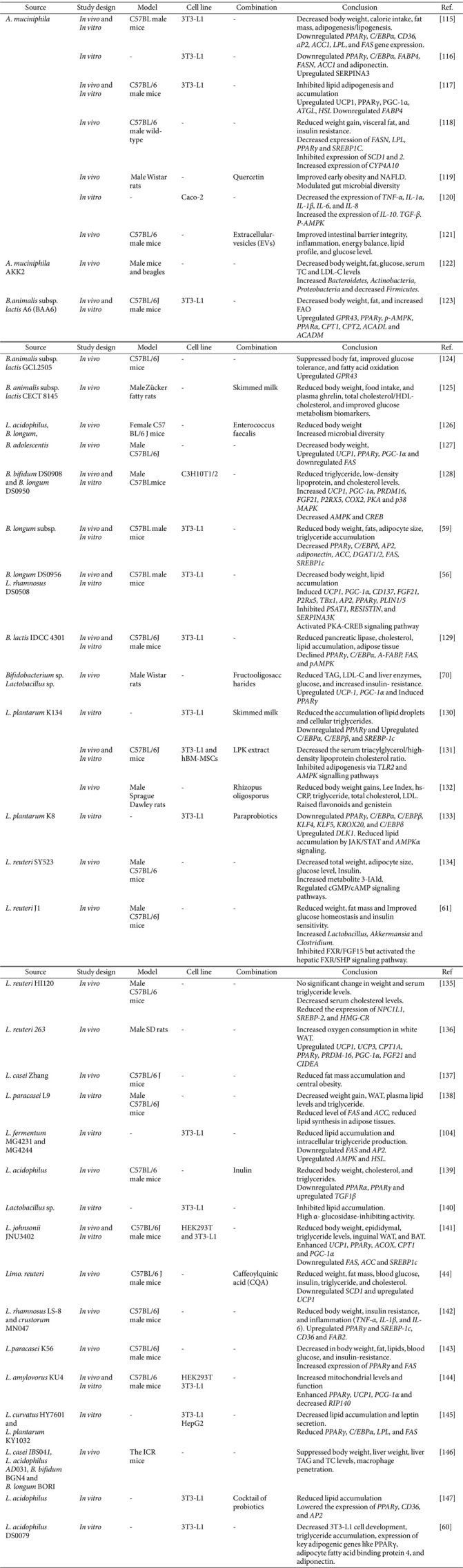

**Table 3:** Insights from clinical studies indicate the role of microbiome in the anti-obesity.

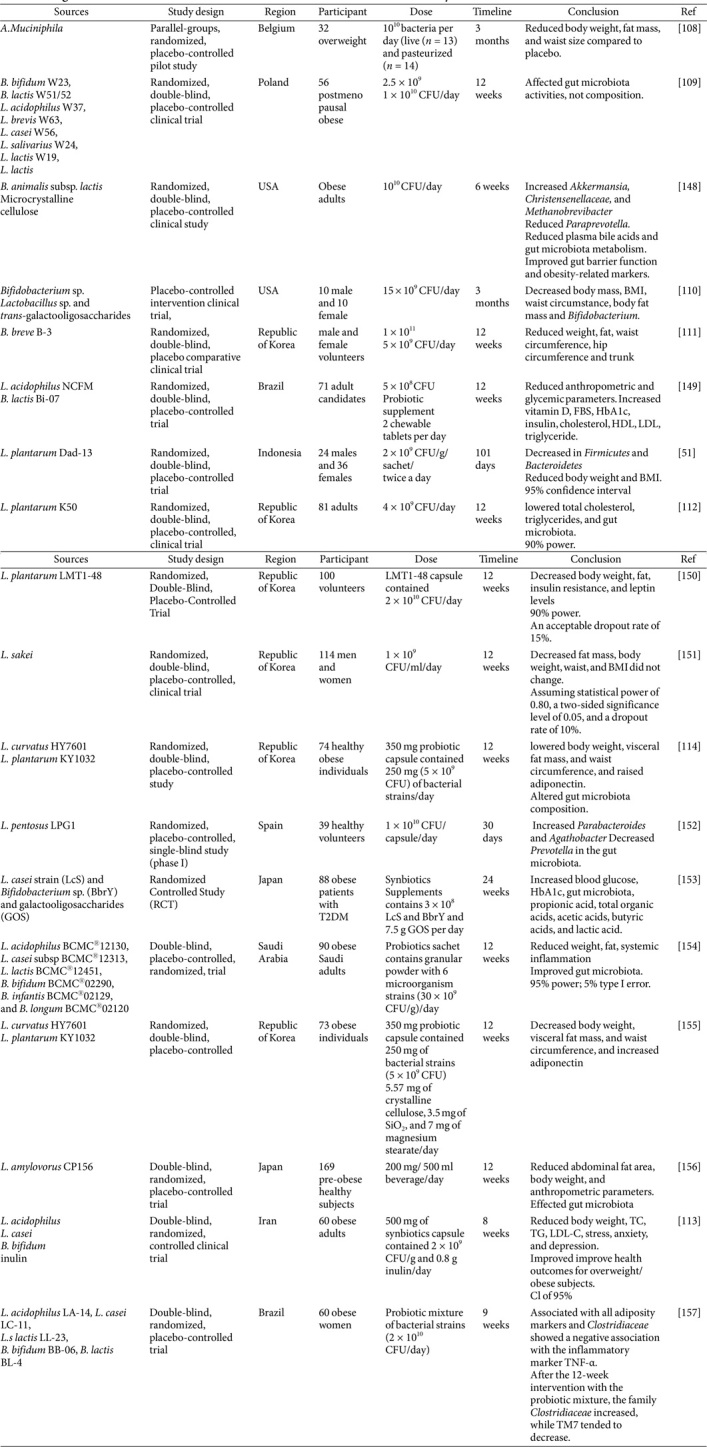

**Table:** Box 1. Taxonomic classification of microbes mentioned in this study.